# Daily mobility, activity and environmental determinants of stress in ecological momentary assessment (EMA) and GPS studies: a scoping review protocol

**DOI:** 10.1136/bmjopen-2024-091509

**Published:** 2025-06-27

**Authors:** Noemie Topalian, Guy Fagherazzi, Camille Perchoux

**Affiliations:** 1 Urban Development and Mobility, Luxembourg Institute of Socio-Economic Research, Esch-sur-Alzette, Esch-sur-Alzette, Luxembourg; 2 Department of Precision Health, Luxembourg Institute of Health, Strassen, Luxembourg District, Luxembourg

**Keywords:** Stress, Psychological, Stress, Physiological, PUBLIC HEALTH

## Abstract

**Abstract:**

**Introduction:**

Stress is omnipresent in our everyday lives and a key risk factor for our physical and mental health. Yet little is known about the impact of geographic life environments, linked to our daily activities and mobility patterns, on our momentary and daily stress levels.

We propose this review to gather evidence on the spatio-temporal determinants of momentary or daily stress in studies using ecological momentary assessment (EMA) or experience sampling methods (ESM) in addition to global positioning systems (GPS) tracking. We will focus on the spatio-temporal definition and modelling of environmental exposures accounting for participant daily activities and mobility patterns and their association with stress.

**Methods and analysis:**

This scoping review will follow the Preferred Reporting Items for Systematic Reviews and Meta-Analyses framework for scoping reviews (2018). We will search the PubMed/Medline, Web of Science, PsycInfo and Scopus databases. We will include papers using EMA or ESM and GPS measuring chronic, daily or momentary stress as an outcome; these methods are also referred to as geographically-explicit ecological momentary assessment.

Articles published from January 2000–June 2025 will be screened. Two independent reviewers will screen titles and abstracts to agree on the inclusion of articles. No geographical or population limitation will be imposed.

**Ethics and dissemination:**

This study is a scoping review based on previously published and publicly available literature. It does not involve the collection of primary data, human participants, or the processing of personal or sensitive information. Therefore, ethical approval is not required in accordance with institutional and international research ethics guidelines. The results will be submitted in peer-reviewed journals and presented at international conferences.

**STRENGTHS AND LIMITATIONS OF THE STUDY**

- This review will identify research gaps in the study of environmental determinants of stress, measured on psychological or physiological levels.
- We will particularly focus on mobility patterns, mobility-related exposures and activity factors in relation to stress.
- This review will identify differences in methodological strategies in ecological momentary assessment/experience sampling methods and global positioning systems studies.
- We will not be able to give comprehensive evidence on the determinants of stress, as studies are very diverse in terms of research questions, methodologies, target population or geographical areas.

## Introduction

 The WHO defines stress as a ‘state of worry or mental tension caused by a difficult situation’.^1^ Understanding which social and geographical contexts (place-based factors, mobility and activity) are associated with stress is essential to reducing people’s exposure to them. Stress is estimated to be a risk factor in 75–90% of diseases and is associated with diabetes, hypertension and cancer among others.^2^

Environmental determinants of stress are various and can range from natural spaces (ie, green spaces, blue spaces) to built environments (ie, architecture, amenities) or sociodemographic characteristics (ie, gender, socio-economic status). In 2016, environmental conditions were estimated to account for 24% of global deaths.^3^

While residential-based longitudinal studies^4^ have widely assessed environmental determinants; they fall under the uncertain geographic context problem^5^ and don’t allow to characterize the exposure outside of home. Ecological momentary assessment (EMA), also referred to as experience sampling methods (ESM) or ambulatory assessment, consists of the repeated measurement of an outcome, over the course of the day.^6^ Coupled with global positioning systems (GPS), it improved the assessment of exposure by recording information on the context at all times.^7^ As 69% of the worldwide population is now equipped with a smartphone, the interest in these studies has grown.^8^

In the case of stress, EMA/ESM and GPS studies are particularly interesting as it fluctuates over the course of the day. These studies precisely measure the exposure of participants to their daily environments and report in real-time social interactions and activities with a very limited recall bias.^6^ Also referred to as geographically-explicit ecological momentary assessment (GEMA), they differ in duration, number of questions, number of prompts and questionnaires. Several reviews have shown their heterogeneity in terms of reporting strategies,^9^ methodologies^10^ and even expositions studied, in the case of mood for instance.^11^

This review aims to (1) Report the methodologies used in GEMA studies for stress, (2) Assess the effect of environmental factors on stress and identify the overlooked determinants in those studies and (3) Investigate the activity and mobility components of GEMA studies on stress.

## Methods and analysis

Our review will follow the Arksey and O’Malley six-stage methodological framework and we will report our results in accordance with the Preferred Reporting Items for Systematic Reviews and Meta-Analyses (PRISMA) framework for scoping reviews.^12 13^ The review started in June 2024 and is planned to end in November 2025.

The stages are the following: (1) Identifying clear research questions, (2) Assessing the feasibility of the review, (3) Selecting relevant studies, (4) Extracting data, (5) Reporting results and (6) Discussion with stakeholders (optional).

### Stage 1: research questions

The research questions are the following:

1. What are the methodologies in EMA/ESM and GPS studies with regard to their design, assessment of stress, mobility and exposure measurement and data analysis?
2. Which environments have a positive or negative effect on stress and which environments are less studied?
3. How are mobility and activity taken into consideration, and what are their roles in the environment-stress relationship?

### Stage 2: feasibility of the review

This stage consists in identifying relevant studies to answer our research questions. Using the Population, Exposure, Outcome, Study design (PEOS) framework was most relevant to identify the extent of our research.^14^

For this review, we do not have any restrictions on the population studied. The purpose of the review is to gather studies assessing any type of stress in repeated measures.

**Table:** 

Population	All human populations
Exposure	Environmental factors, place-based factors, mobility and activity types
Outcome	Stress
Study design	Ecological momentary assessment paired with GPS tracking

### Stage 3: selection of the studies

We will search the PubMed/Medline, Web of Science, PsycInfo and Scopus databases from January 2000–June 2025.

#### Inclusion criteria

- We include all populations (all ages, genders, mental or physical health conditions).
- We will include all environmental determinants (natural, built and social environments).
- We will include all assessments of stress (psychological and physiological measures).
- We include only studies with EMA/ESM and GPS in their study design. Specifically, we exclude self-reports of locations or environmental context if not complement GPS data.
- We will include study protocols and pilot studies.

#### Exclusion criteria

- We will not include grey or unpublished literature, books and non-peer-reviewed publications.
- We will exclude other mental health outcomes different from stress (well-being, depression, schizophrenia, Post-traumatic stress disorder) and focus on chronic, momentary and daily stress.
- We will exclude studies not written in English.

The complete list of search terms is available in the online supplemental file 1. We will use the CADIMA tool to conduct this review.^15^ Two independent reviewers (NT, CP) will perform screening, and consistency checks will be carried out with CADIMA.

The first step consists of screening titles and abstracts to do a first selection of eligible studies. The remaining will be full-text screened by both reviewers who will discuss articles where the agreement was not met.

### Stage 4: data extraction

This step consists of charting the data. To reduce potential errors and biases, data extraction will be performed by a primary reviewer (NT) and a second reviewer (CP) will extract data from a random subset of 20% of included studies. Both reviewers will discuss the inconsistencies, and we will report the inter-rater reliability.

The following elements will be extracted, in accordance with the STROBE-GEMA reporting guidelines:^9^

- Author.
- Journal and year of publication.
- Country/region.
- Type of study (Observational cohort, Randomised controlled trial…).
- Research questions.
- Study population.
- Baseline assessment.
- Environmental factors studied.
- Mobility and mobility-related exposure definition/measurements.
- Activity factors studied.
- Stress measurement.
- EMA/ESM and GPS design (number of days, number of prompts, sampling rate, missing data, compliance, number of questions).
- Methods for the analysis.
- Key findings.
- Limitations.

### Stage 5: reporting the results

On the one hand, we will report our findings on each study regarding methodologies and the environmental factors investigated. We will also report the year and location. We will compare the methodologies by duration of the studies and frequency of EMA prompts and see how the measures of stress differ between studies.

On the other hand, we will divide the evidence on the determinants of stress into several parts:

- Place-based factors (ie, natural, built environments).
- Socio-demographic factors (ie, socioeconomic status, social disorganisation).
- Activity factors.
- Mobility factors.

We will report the exposure and environmental measures used in each study. We will also investigate the interactions between those factors. We will report these results in a narrative way and summarise the extracted data into tables (methodology and environmental factors). We will address the individual and common limitations found in these studies. Our discussion should open reflections on EMA/ESM and GPS studies and give insights for future studies on the integration of mobility and activity components.

## Discussion

We acknowledge that this review will have several strengths and limitations. First, this review will encompass a large number of search terms to include all the studies targeted. We anticipate that selecting the studies will be a challenge due to the various names given to GEMA studies. We will also cover all measures of stress. The findings of this study will allow us to provide methodological recommendations for future GEMA studies on stress and give insights into the environmental influences of stress.

We will only include literature published after the year 2000. As GPS-enabled devices and smartphones became widely available in the early 2000s, it would not be methodologically relevant to include studies prior to this date. Only publications written in English were included in this review to ensure accuracy in the data extraction and interpretation of the study contents. We acknowledge this as a limitation as it could introduce a language bias.

## Patient and public involvement

None.

## Ethics and dissemination

This study is a scoping review based on previously published and publicly available literature. It does not involve the collection of primary data, human participants or the processing of personal or sensitive information. Therefore, ethical approval is not required in accordance with institutional and international research ethics guidelines.^16^

The results will be submitted in peer-reviewed journals and presented at international conferences. All data gathered from this study (ie, extracted from included articles) will be made publicly available. This review will give recommendations for future stress studies using EMA/ESM and GPS in terms of study design and understudied determinants.

## Supplementary material

10.1136/bmjopen-2024-091509 online supplemental file 1
