# Supplementary material for: Daily mobility, activity and environmental determinants of stress in ecological momentary assessment (EMA) and GPS studies: a scoping review protocol
Source: BMJ Open. 2025 Jun 27;15(6):e091509. doi: 10.1136/bmjopen-2024-091509 (PMC12207153; doi:10.1136/bmjopen-2024-091509)
Supplement: online supplemental file 1 [file bmjopen-15-6-s001.docx]

**ANNEX**

In PubMed/Medline

(stress*[Title/Abstract] OR mental* health*[Title/Abstract] OR psychological* health*[Title/Abstract] OR mood[Title/Abstract] OR wellbeing[Title/Abstract] OR well-being[Title/Abstract] OR affect[Title/Abstract] OR anxiety[Title/Abstract] OR cortisol[Title/Abstract] OR heart rate[Title/Abstract] OR electrodermal activity[Title/Abstract] OR SCL[Title/Abstract] OR SCR[Title/Abstract]) AND (EMA[Title/Abstract] OR ESM[Title/Abstract] OR daily diary[Title/Abstract] OR experience sampling[Title/Abstract] OR ecological momentary assessment[Title/Abstract] OR event sampling[Title/Abstract] OR daily measure*[Title/Abstract] OR mobile survey[Title/Abstract] OR momentary assessment[Title/Abstract] OR ambulatory assessment[Title/Abstract] OR digital phenotyping[Title/Abstract]) AND (GPS[Title/Abstract] OR global positioning system[Title/Abstract] OR location*[Title/Abstract] OR mobil*[Title/Abstract] OR GMA[Title/Abstract] OR GEMA[Title/Abstract] OR geographic* momentary assessment[Title/Abstract] OR transport*[Title/Abstract] OR movement[Title/Abstract] OR spatio-temporal[Title/Abstract] OR spatiotemporal[Title/Abstract] OR smartphone tracking[Title/Abstract] OR non residential[Title/Abstract]) NOT (general practitioners) AND (environment*[Title/Abstract] OR green space[Title/Abstract] OR greenspace[Title/Abstract] OR exposure*[Title/Abstract] OR neighborhood[Title/Abstract] OR neighbourhood[Title/Abstract] OR blue space[Title/Abstract] OR stressor*[Title/Abstract] OR traffic[Title/Abstract] OR noise[Title/Abstract] OR pollution[Title/Abstract] OR light[Title/Abstract] OR architecture[Title/Abstract] OR context[Title/Abstract] OR park[Title/Abstract] OR greenness[Title/Abstract] OR vegeta*[Title/Abstract] OR trees[Title/Abstract] OR land use[Title/Abstract] OR landuse[Title/Abstract] OR urban*[Title/Abstract] OR built[Title/Abstract] OR city[Title/Abstract] OR street[Title/Abstract] OR landscape[Title/Abstract] OR rural[Title/Abstract] OR natur*[Title/Abstract] OR bird[Title/Abstract] OR place based factor[Title/Abstract] OR facilit*[Title/Abstract] OR amenit*[Title/Abstract] OR densit*[Title/Abstract] OR walkability[Title/Abstract] OR activit*[Title/Abstract])

Filters: Start date=2000

568 results, 5th September 2024

In Web of Science

(TS=(stress*) OR TS=(“mental* health*”) OR TS=(“psychological* health*”) OR TS=(mood) OR TS=(wellbeing) OR TS=(well-being) OR TS=(affect) OR TS=(anxiety) OR TS=(cortisol) OR TS=(“heart rate”) OR TS=(“electrodermal activity”) OR TS=(SCL) OR TS=(SCR)) AND (TS=(EMA) OR TS=(ESM) OR TS=(“daily diary”) OR TS=(“experience sampling”) OR TS=(“ecological momentary assessment”) OR TS=(“event sampling”) OR TS=(“daily measure*”) OR TS=(“mobile survey”) OR TS=(“momentary assessment”) OR TS=(“ambulatory assessment”) OR TS=(“digital phenotyping”)) AND (TS=(GPS) OR TS=(“global positioning system”) OR TS=(location*) OR TS=(mobil*) OR TS=(GMA) OR TS=(GEMA) OR TS=(“geographic* momentary assessment”) OR TS=(transport*) OR TS=(movement) OR TS=(spatio-temporal) OR TS=(spatiotemporal) OR TS=(“smartphone tracking”) OR TS=(“non residential”)) AND (TS=(environment*) OR TS=(“green space”) OR TS=(greenspace) OR TS=(exposure*) OR TS=(neighborhood) OR TS=(“blue space”) OR TS=(stressor*) OR TS=(traffic) OR TS=(noise) OR TS=(pollution) OR TS=(light) OR TS=(architecture) OR TS=(context) OR TS=(park) OR TS=(greenness) OR TS=(vegeta*) OR TS=(trees) OR TS=(“land use”) OR TS=(landuse) OR TS=(urban*) OR TS=(built) OR TS=(city) OR TS=(street) OR TS=(landscape) OR TS=(rural) OR TS=(natur*) OR TS=(bird) OR TS=(“place based factor”) OR TS=(facilit*) OR TS=(amenit*) OR TS=(densit*) OR TS=(walkability) OR TS=(activit*)) NOT TS=(“general practitioners”)

Filters: Publication date between 2000-01-01 and 2024-09-05, search in the Web of Science Core Collection, All Editions

1074 results, 5th September 2024

In Scopus

( TITLE-ABS-KEY ( stress* OR mental* AND health* OR psychological* AND health* OR mood OR anxiety OR wellbeing OR well-being OR affect OR cortisol OR "heart rate" OR "electrodermal activity" OR scl OR scr ) AND TITLE-ABS-KEY ( ema OR esm OR "daily diary" OR "experience sampling" OR "ecological momentary assessment" OR "event sampling" OR "daily measure*" OR "mobile survey" OR "momentary assessment" OR "ambulatory assessment" OR "digital phenotyping" ) AND TITLE-ABS-KEY ( gps OR "global positioning system" OR location* OR mobil* OR gma OR gema OR "geographic* momentary assessment" OR transport* OR movement OR spatio-temporal OR spatiotemporal OR "smartphone tracking" OR "non residential" ) AND NOT ALL ( "general practitioners" ) AND TITLE-ABS-KEY ( environment* OR "green space" OR greenspace OR exposure* OR neighborhood OR neighbourhood OR "blue space" OR stressor* OR traffic OR noise OR pollution OR light OR architecture OR context OR park OR greenness OR vegeta* OR trees OR "land use" OR landuse OR urban* OR built OR city OR street OR landscape OR rural OR natur* OR bird OR "place based factor" OR facilit* OR amenit* OR densit* OR walkability OR activit*) ) AND PUBYEAR > 1999

Filters: Publication year after 1999 (in query)

784 results, 5th September 2024

In psycInfo

((((stress* or mental* health* or psychological* health* or mood or anxiety or well?being or affect or cortisol or heart rate or electrodermal activity or SCL or SCR) and (EMA or ESM or daily diary or experience sampling or event sampling or daily measure* or mobile survey or momentary assessment or ambulatory assessment or digital phenotyping) and (GPS or global positioning system or location* or mobil* or GMA or GEMA or geographic* momentary assessment or transport* or movement or spatio?temporal or smartphone tracking or non residential)) not general practitioners) and (environment* or green?space or exposure* or neighbo?rhood or blue space or stressor* or traffic or noise or pollution or light or architecture or context or park or greenness or vegeta* or trees or land?use or urban* or built or city or street or landscape or rural or natur* or bird or place based factor or facilit* or amenit* or densit* or walkability or activit*)).mp. [mp=title, abstract, heading word, table of contents, key concepts, original title, tests & measures, mesh word]

Filters: year 2000 to current

444 results, 5th September 2024

The search will be updated for all databases from September 2024 until June 2025.
