# Supplementary material for: Daily mobility, activity and environmental determinants of stress in ecological momentary assessment (EMA) and GPS studies: a scoping review protocol
Source: BMJ Open. 2025 Jun 27;15(6):e091509. doi: 10.1136/bmjopen-2024-091509 (PMC12207153; doi:10.1136/bmjopen-2024-091509)
Supplement: Supplementary file 2 [file bmjopen-2024-091509.reviewer_comments.pdf]

PEER REVIEW HISTORY

BMJ Open publishes all reviews undertaken for accepted manuscripts. Reviewers are asked to complete a checklist review form and are provided with free text boxes to elaborate on their assessment. These free text comments are reproduced below.

ARTICLE DETAILS

Title (Provisional)

Daily Mobility, Activity and Environmental Determinants of Stress in Ecological Momentary Assessment (EMA) and GPS Studies: A Scoping Review Protocol

Authors

Topalian, Noemie; Fagherazzi, Guy; Perchoux, Camille

VERSION 1 - REVIEW

**Reviewer**	**1**
**Name**	**Wray, Alexander**
**Affiliation**	**Western University**
**Date**	**12-Nov-2024**
**COI**	**None**

The authors propose a scoping review protocol to examine how geographic ecological momentary assessment methods have been used to evaluate external determinants of stress. The protocol is well-described, however, I would provide the following feedback to further strengthen their proposed review:

The topical scope of the scoping review is considerably narrow, though still valuable. Given the narrow scope, I think the authors are describing a systematic review rather than a scoping review. Both geographic ecological momentary assessment and stress as an outcome in the environmental determinants of health are clearly defined in the literature. A scoping review is typically used to further refine a topical area for later reviews. There have already been multiple scoping reviews of GEMA and the environmental determinants of health, that would suggest this be classified as a systematic review.

I would ask the authors to confirm they will extract in Stage 4 all the reporting elements that are contained within the recently published STROBE-GEMA guidance material found in Kingsbury C, Buzzi M, Chaix B, Kanning M, Khezri S, Kiani B, Kirchner TR, Maurel A, Thierry B, Kestens Y. STROBE-GEMA: a STROBE extension for reporting of geographically explicit ecological momentary assessment studies. Archives of Public Health. 2024 Jun 13;82(1):84.

The search terms are appropriate for the study, however, I would encourage the authors to test multiple combinations of Boolean statements to ensure there are no inadvertent exclusions. The authors may also wish to consider adding “experiential” to the second group of terms, and “mobility” to the third group of terms.

**Reviewer**	**2**
**Name**	**Hinwood, Madeleine**
**Affiliation**	**The University of Newcastle, School of Medicine and Public Health**
**Date**	**06-May-2025**
**COI**	**None**

Intro: You cite the WHO definition of stress as a 'state of worry or mental tension caused by a difficult situation' (1), but the second half of the first sentence is vague. This then fails to adequately contextualise the focus of the study- what constitutes 'difficult situations' in the research framework?

You state that 'residential-based longitudinal studies (4) have widely assessed environmental determinants, the use of EMA or ESM and GPS studies, enabled by smartphones, improved the assessment of exposure to stressors.' The claim that these study designs improve the assessment of exposure to stressors needs a citation. Additionally, you should redefine the acronyms at this point in the text, justify why these methodologies can be grouped together, and articulate the unique contributions to stress research these can make, compared to traditional/other methods- this section needs expanding. I am also not sure what is meant by residential studies.

The paragraph beginning with 'Several reviews have been published...' lacks a clear central argument. The paragraph moves between discussing existing reviews, methodological components, and general advantages of EMA/ESM studies without establishing logical connections between these points. This paragraph should be restructured to present a single, clear idea with supporting details that build toward the paper's research aims.

You state [as the overall research aim]: 'We aim to identify gaps in determinants and methodological differences in these studies. To our knowledge, no review has investigated the activity and mobility components of GEMA studies on stress.' This aim does not logically follow from the preceding introduction, which fails to establish why identifying these gaps is important or necessary. The phrase 'gaps in determinants' is ambiguous—do you mean the ability of these methods to pick up overlooked environmental factors, methodological limitations in measuring known determinants, or something else? The research gap and significance of the study should be more precisely articulated.

Similarly, the research questions presented do not align clearly with the stated aim of identifying 'gaps in determinants and methodological differences.' Research question 1 addresses methodological aspects, which partially aligns with the aim. However, questions 2 and 3 focus on empirical findings about environment-stress relationships and mobility/activity influences, rather than gaps or methodological differences. Please consider either revising your aim statement to encompass these broader questions or refocus the research questions to better address the stated aim of the review.

The inclusion and exclusion criteria should be clearly separated into distinct sections with appropriate subheadings.

There is an inconsistency between the exclusion criteria and the included sample search strategy. While you state that you exclude other mental health outcomes different from stress (well-being, depression, schizophrenia, PTSD) and focus on chronic, momentary and daily stress,' your search strategy includes terms explicitly related to these excluded mental health conditions. This discrepancy needs to be resolved.

The PICOS framework is possibly not appropriate for this type of review. Given the observational nature of the included studies, you could consider adopting an alternatives- maybe the PEO/PEOS (Population, Exposure, Outcome +/- study design) framework instead- you need a framework which will better accommodate the research questions and types of studies being reviewed.

Data extraction is the be performed by a single reviewer, which introduces potential risk of bias and error. Please consider implementing a dual-review process where two independent reviewers extract data from a substantial subset of studies (e.g., 20%) to establish inter-rater reliability and validate the data charting form.

The 'Reporting the Results' section lacks specificity regarding data synthesis methods. While you outline broad categories for organising findings, you have not included details such as how data will be summarised, compared, and integrated across studies. Please briefly specify your approach to data synthesis (e.g., narrative synthesis with tabular summaries) and explain how you might handle heterogeneity across studies in terms of methods, measures, and findings.

Please consider adding a Discussion section that addresses anticipated contributions, potential challenges, and limitations of the proposed review. This could include some information on how findings might advance theoretical understanding of environmental influences on stress, inform methodological improvements in these types of studies, and/or guide future research directions.

VERSION 1 - AUTHOR RESPONSE

Reviewer: 1

Dr. Alexander Wray, Western University

The authors propose a scoping review protocol to examine how geographic ecological momentary assessment methods have been used to evaluate external determinants of stress. The protocol is well-described, however, I would provide the following feedback to further strengthen their proposed review.
The topical scope of the scoping review is considerably narrow, though still valuable. Given the narrow scope, I think the authors are describing a systematic review rather than a scoping review. Both geographic ecological momentary assessment and stress as an outcome in the environmental determinants of health are clearly defined in the literature. A scoping review is typically used to further refine a topical area for later reviews. There have already been multiple scoping reviews of GEMA and the environmental determinants of health, that would suggest this be classified as a systematic review.

Our response:
Thank you for your insightful comments and feedback. We agree on the fact that the topic is narrow, especially as per the outcome (stress). However, we chose to define our review as scoping for two reasons. First, the environmental determinants of health are broad, and one of our aims is to identify gaps in the search for those factors, which fits more into the scoping category. Secondly, we identified on Prospero (CRD42023432842) a systematic review on the environmental determinants of mental health, covering the topic of stress. Therefore, we decided to broaden our review to mobility and activity aspects in order to bring added value and novelty to this review.

I would ask the authors to confirm they will extract in Stage 4 all the reporting elements that are contained within the recently published STROBE-GEMA guidance material found in Kingsbury C, Buzzi M, Chaix B, Kanning M, Khezri S, Kiani B, Kirchner TR, Maurel A, Thierry B, Kestens Y. STROBE-GEMA: a STROBE extension for reporting of geographically explicit ecological momentary assessment studies. Archives of Public Health. 2024 Jun 13;82(1):84.

Our response:
Thank you for your suggestion. We will indeed follow the STROBE-GEMA reporting guidelines regarding the GEMA methods (number of days, prompts, compliance etc.), technology, measure of the outcome (stress) and exposure. We included this precision in the Stage 4 of the Methods section "The following elements will be extracted, in accordance with the STROBE-GEMA reporting guidelines [9]".

The search terms are appropriate for the study, however, I would encourage the authors to test multiple combinations of Boolean statements to ensure there are no inadvertent exclusions. The authors may also wish to consider adding "experiential" to the second group of terms, and "mobility" to the third group of terms.

Our response:
Thank you, we agree with your suggestion. We have tested multiple combinations of keywords (mostly switching "activity" into the 3rd group and adding "mobility" into the 4th, and adding "repeated measure" into the 2nd) but these combinations were retrieving too many results that were out of scope, of studies not using GPS. "Mobility" is already included in the 3rd group of terms as "mobil*" so encompass all its suffixes (mobility, mobile). A quick search showed that adding "experiential" did not retrieve many more results.

<u>Reviewer: 2</u>
Dr. Madeleine Hinwood, The University of Newcastle

Intro: You cite the WHO definition of stress as a 'state of worry or mental tension caused by a difficult situation' (1), but the second half of the first sentence is vague. This then fails to adequately contextualise the focus of the study- what constitutes 'difficult situations' in the research framework?

<u>Our response:</u>
Thank you for your remark. We have changed the second half of the sentence to better frame the focus of the study: “The World Health Organization defines stress as a “state of worry or mental tension caused by a difficult situation” [1]. Understanding which social and geographical contexts (place-based factors, mobility and activity) are associated with stress is essential to reduce people’s exposure to them.”

You state that 'residential-based longitudinal studies (4) have widely assessed environmental determinants, the use of EMA or ESM and GPS studies, enabled by smartphones, improved the assessment of exposure to stressors.' The claim that these study designs improve the assessment of exposure to stressors needs a citation. Additionally, you should redefine the acronyms at this point in the text, justify why these methodologies can be grouped together, and articulate the unique contributions to stress research these can make, compared to traditional/other methods- this section needs expanding. I am also not sure what is meant by residential studies.

<u>Our response:</u>
Thank you for your suggestion. We have clarified this paragraph and added the necessary justifications: “While residential-based longitudinal studies [4] have widely assessed environmental determinants, they fall under the uncertain geographic context problem [5] and don’t allow to determine the exposure outside of home. Ecological Momentary Assessment (EMA), also referred to as Experience Sampling Methods (ESM) or ambulatory assessment, consists in the repeated measurement of an outcome, over the course of the day [6]. Coupled with Global Positioning Systems (GPS), it improved the assessment of exposure by recording information on context at all times [7]. As 69% of the worldwide population is now equipped with a smartphone, the interest for these studies has grown [8].”

The paragraph beginning with 'Several reviews have been published...' lacks a clear central argument. The paragraph moves between discussing existing reviews, methodological components, and general advantages of EMA/ESM studies without establishing logical connections between these points. This paragraph should be restructured to present a single, clear idea with supporting details that build toward the paper's research aims.

<u>Our response:</u>
Thank you. As for the last paragraph, we restructured and detailed it more as suggested: “In the case of stress, EMA/ESM and GPS studies are particularly interesting as it fluctuates over the course of the day. These studies precisely measure the exposure of participants to their daily environments and report in real-time social interactions and activities with a very limited recall bias [6]. Also referred to as GEMA, they differ in duration, number of questions, number of prompts and questionnaires. Several reviews have shown their heterogeneity in terms of reporting strategies [9], methodologies [10] and even expositions studied, in the case of mood for instance [11].”

You state [as the overall research aim]: 'We aim to identify gaps in determinants and methodological differences in these studies. To our knowledge, no review has investigated the activity and mobility components of GEMA studies on stress.' This aim does not logically follow from the preceding introduction, which fails to establish why identifying these gaps is important or necessary. The phrase 'gaps in determinants' is ambiguous—do you mean the ability of these methods to pick up overlooked environmental factors, methodological limitations in measuring known determinants, or something else? The research gap and significance of the study should be more precisely articulated.
Similarly, the research questions presented do not align clearly with the stated aim of identifying 'gaps in determinants and methodological differences.' Research question 1 addresses methodological aspects, which partially aligns with the aim. However, questions 2 and 3 focus on empirical findings about environment-stress relationships and mobility/activity influences, rather than gaps or methodological differences. Please consider either revising your aim statement to encompass these broader questions or refocus the research questions to better address the stated aim of the review.

Our response:
Thank you for your remark. We have clarified the global aims of the review in the introduction "This review aims to (1) report the methodologies used in GEMA studies for stress; (2) assess the effect of environmental factors on stress and identify the overlooked determinants in those studies; (3) investigate the activity and mobility components of GEMA studies on stress." Consequently, we also reviewed our research question n°2 to clarify its aims: "Which environments have a positive or negative effect on stress and which environments are less studied?"

The inclusion and exclusion criteria should be clearly separated into distinct sections with appropriate subheadings.

Our response:
We agree with your remark, we have now separated both sections.

There is an inconsistency between the exclusion criteria and the included sample search strategy. While you state that you exclude other mental health outcomes different from stress (well-being, depression, schizophrenia, PTSD) and focus on chronic, momentary and daily stress,' your search strategy includes terms explicitly related to these excluded mental health conditions. This discrepancy needs to be resolved.

Our response:
Thank you for your comment. We have selected broader outcomes in the search terms and included psychological health, mood and anxiety because they are sometimes used as an umbrella term and their sub-dimensions might encompass stress. We will look at these studies individually and include them if they report a separate measure of stress.

The PICOS framework is possibly not appropriate for this type of review. Given the observational nature of the included studies, you could consider adopting an alternatives- maybe the PEO/PEOS (Population, Exposure, Outcome +/- study design) framework instead- you need a framework which will better accommodate the research questions and types of studies being reviewed.

Our response:
Thank you for your suggestions and providing us with alternatives. We changed the framework to PEOS which indeed better fits the type of studies and our research questions: "Using the PEOS framework was most relevant to identify the extent of our research [14]".

Data extraction is to be performed by a single reviewer, which introduces potential risk of bias and error. Please consider implementing a dual-review process where two independent reviewers extract data from a substantial subset of studies (e.g., 20%) to establish inter-rater reliability and validate the data charting form.

Our response:
We agree with you comment. We will implement a dual-review process as suggested: "To reduce potential errors and biases, data extraction will be performed by a primary reviewer (NT) and a second reviewer (CP) will extract data from a random subset of 20% of included studies. Both reviewers will discuss the inconsistencies and we will report the inter-rater reliability."
The 'Reporting the Results' section lacks specificity regarding data synthesis methods. While you outline broad categories for organising findings, you have not included details such as how data will be summarised, compared, and integrated across studies. Please briefly specify your approach to data synthesis (e.g., narrative synthesis with tabular summaries) and explain how you might handle heterogeneity across studies in terms of methods, measures, and findings.

Our response:
Thank you for your remark. We have added precisions on how the data will be compared: "On the one hand, we will report our findings on each study regarding methodologies and the environmental factors investigated. We will also report the year and location. We will compare the methodologies by duration of the studies, frequency of EMA prompts and see how the measures of stress differ between studies."

We have also clarified how we will organize our findings: "We will report the exposure and environmental measures used in each study. We will also investigate the interactions between those factors. We will report these results in a narrative way and summarize the extracted data into tables (methodology and environmental factors)."

Please consider adding a Discussion section that addresses anticipated contributions, potential challenges, and limitations of the proposed review. This could include some information on how findings might advance theoretical understanding of environmental influences on stress, inform methodological improvements in these types of studies, and/or guide future research directions.

Our response:
Thank you for your suggestion. We have added a Discussion section addressing the aims of the review and limitations of our study:
"We acknowledge that this review will have several strengths and limitations. First, this review will encompass a large number of search terms to include all the studies targeted. We anticipate that selecting the studies will be a challenge due to the various names given to GEMA studies. We will also cover all measures of stress. The findings of this study will allow us to provide methodological recommendations for future GEMA studies on stress and give insights on the environmental influences of stress.

We will only include literature published after the year 2000. As GPS-enabled devices and smartphones became widely available in the early 2000s, it would not be methodologically relevant to include studies prior to this date. Only publications written in English were included in this review to ensure accuracy in the data extraction and interpretation of the study contents. We acknowledge this as a limitation as it could introduce a language bias.”
